# Supplementary material for: Scrub typhus in pregnancy: A 10-year multicenter study in resource-limited settings in China
Source: PLoS Negl Trop Dis. 2025 Jan 21;19(1):e0012829. doi: 10.1371/journal.pntd.0012829 (PMC11771934; doi:10.1371/journal.pntd.0012829)
Supplement: Attachment — Submitted filename: Response to Reviewers-PNTD-D-24-01393R1.pdf [file pntd.0012829.s002.pdf]

Response to Reviewers

Manuscript #: PNTD-D-24-01393
Title: Scrub typhus in pregnancy: A 10-year multicenter study in resource-limited settings in China

Please find below a point-by-point response to the reviewer concerns. Based on the reviewers' comments, we have made revision which marked in different colors (deletion in red, addition in yellow) in the file labeled 'Revised Manuscript with Track Changes-PNTD-D-24-01393'.

Reviewers' Comments:
Reviewer's Responses to Questions
Key Review Criteria Required for Acceptance?
As you describe the new analyses required for acceptance, please consider the following:

Methods
-Are the objectives of the study clearly articulated with a clear testable hypothesis stated?
-Is the study design appropriate to address the stated objectives?
-Is the population clearly described and appropriate for the hypothesis being tested?
-Is the sample size sufficient to ensure adequate power to address the hypothesis being tested?
-Were correct statistical analysis used to support conclusions?
-Are there concerns about ethical or regulatory requirements being met?

Reviewer #1: Objective clear, study design-appropriate, statastics correctly used
Reply: Thank you for the positive comments.

Reviewer #2:
Reply: Thank you for all the thoughtful and informative reviews. I really appreciate it.

1. Please provide the day of illness on which treatment was begun.
Reply: Thank you for your comments.
The median (IQR) time from the onset of fever to the definitive diagnosis of scrub typhus (ST) was 4 (3-5) days. Once ST was diagnosed, antibiotic therapy for ST was commenced.
We added this information in lines 189-191 in the file labeled 'Revised Manuscript with Track Changes'.

2. A single titer of 1:80 of Proteus OXK agglutination is not specific. What is the incidence of titer of 1:80 in healthy persons in Yunnan? As recognized by the authors, Weil Felix serology is not specific. A fourfold rise or greater in titer would be supportive of the diagnosis for purposes of this study. According to the diagnostic criteria, exposure to a disease endemic area, rash, and a titer of 1:80 would be confirmatory of the

diagnosis. This is not sufficient. Many diseases could meet these criteria.

Reply: Thank you for your comments.

Sorry, we didn't find studies that reported the incidence of titer of 1:80 in healthy persons in Yunnan. In clinical practice, Weil-Felix test is usually used to diagnose whether patients had rickettsial infections in Yunnan. In fact, healthy persons are unlikely to do Weil-Felix test.

We agree that a fourfold rise or greater in titer would be more supportive of the diagnosis than a titer of 1:80. Based on your comments, we revised the laboratory diagnostic criteria: A minimum titer of 1:160 or a fourfold increase over previous levels for OXK is considered diagnostically significant. Please find the revision in line 155 in the file labeled 'Revised Manuscript with Track Changes-PNTD-D-24-01393'.

Based on the revised laboratory diagnostic criteria, we reanalyzed the Weil-Felix test results of all 260 patients. In total, 75.4% (n=196) had Weil-Felix test, in which 52.5% (n=103), 1.0% (n=2) and 46.4% (n=91) had a titer of 1:40, 1:80, and ≥1:160 for OXK, respectively. Please find the revision in lines 185-187 in the file labeled 'Revised Manuscript with Track Changes-PNTD-D-24-01393'.

In addition, patients were separated into clinically diagnosed cases and laboratory confirmed cases. The clinical presentation, treatment, and outcome were compared for patients between clinically diagnosed cases and laboratory confirmed cases. Please find the revision in lines 184-194 and table 1 in the file labeled 'Revised Manuscript with Track Changes-PNTD-D-24-01393'.

3. Table 1 what does "duration of fever, dates" mean? Does this reflect the entire course of illness or the number of days prior to treatment?

Reply: Sorry for the confusion. "duration of fever, days" in Table 1 reflects the number of days that patient had a fever.

4. Tables 1, 2, and 3: What is the difference between "Weil-Felix test" and "positive Weil-Felix test"?

Reply: Thank you for your comments. Sorry for the confusion.

"Weil-Felix test" reflects the number of patients who did this test.

"positive Weil-Felix test" reflects the number of patients who had a positive result: a minimum titer of 1:160 or a fourfold increase over previous levels for OXK.

In order to present the data more clearly, we replaced "Weil-Felix test" with "patients did Weil-Felix test". Please find the revision in Tables 2, 3 and 4 in the file labeled 'Revised Manuscript with Track Changes-PNTD-D-24-01393'.

5. Lines 411 and 412: Please cite a reference documenting congenital anomalies due to vertically transmitted Orientia tsutsugamushi. I do not believe that this occurs.

Reply: Thank you for picking this up. We agree that there was no report showing congenital anomalies due to vertically transmitted Orientia tsutsugamushi.

We replaced the sentence: "Pathogens can be vertically transmitted to the fetus leading to congenital anomalies in 3% of live births and 10-30% of all stillbirths [22-23]. ST was associated with poor fetal outcomes, including miscarriage, intrauterine death,

stillbirth, and preterm delivery [8-14]” with “Pathogens, such as 'TORCH' (Toxoplasma gondii, other, rubella virus, cytomegalovirus, herpes simplex virus), Escherichia coli, group B streptococcus and enterococcus species, can be vertically transmitted to the fetus leading to congenital anomalies in 3% of live births and 10-30% of all stillbirths [22-23]. Although there was no report showing congenital anomalies due to vertically transmitted Orientia tsutsugamushi, ST was associated with poor fetal outcomes, including miscarriage, intrauterine death, stillbirth, and preterm delivery [8-14].
Please find the revision in lines 425-430 in the file labeled 'Revised Manuscript with Track Changes-PNTD-D-24-01393'.

Results
-Does the analysis presented match the analysis plan?
-Are the results clearly and completely presented?
-Are the figures (Tables, Images) of sufficient quality for clarity?

Reviewer #1: All matched
Reply: Thank you for the positive comments.

Reviewer #2: The results should be separated into groups that are documented by seroconversion or by only a single Weil-Felix titer and the presence of an eschar.
The study contains a robust number of cases, the great majority of which manifested eschar and an interesting analysis of the fetal outcomes by trimester.
Reply: Thank you for the suggestion.
Based on your comments, patients were separated into clinically diagnosed cases and laboratory confirmed cases. The clinical presentation, treatment, and outcome were compared for patients between clinically diagnosed cases and laboratory confirmed cases. Please find the revision in lines 184-194 and table 1 in the file labeled 'Revised Manuscript with Track Changes-PNTD-D-24-01393'.
A total of 260 pregnant women were diagnosed with ST, including clinically diagnosed cases 65.0% (n=169) and laboratory confirmed cases 35.0% (n=91) (Table 1). There were no statistically significant differences in antibiotic treatment and fetal outcome, but a higher proportion of patients with pneumonia (18.7% vs. 9.5%, p=0.033) and a longer length of hospitalization (7 vs. 6 days, p=0.007) were seen in laboratory confirmed cases than clinically diagnosed cases.

Table 1 Clinically diagnosed cases Versus laboratory confirmed cases: clinical profile, management and outcome of 260 pregnant women with ST

Diagnosis	Total	clinically diagnosed	laboratory confirmed	P value
N of patients, n (%)	260	169 (65.0)	91 (35.0)	-
Pregnancy in weeks	24 (18-30)	24 (18-32)	24 (19-28)	0.471
First trimester, n (%)	39 (15.0)	24 (14.2)	15 (16.5)	0.623
Second trimester, n (%)	121 (46.5)	76 (45.0)	45 (49.5)	0.490
Third trimester, n (%)	100 (38.5)	69 (40.8)	31 (34.1)	0.285
$T \geq 39°C$, n (%)	165 (63.5)	113 (66.9)	52 (57.1)	0.235
Duration of fever, days	6 (4-8)	6 (5-8)	6 (4-8)	0.453
Eschar, n (%)	204 (78.5)	169 (100)	35 (38.5)	<0.001
Lymphadenopathy, n (%)	10 (3.8)	5 (3.0)	5 (5.5)	0.499
Skin rash, n (%)	7 (2.7)	4 (2.4)	3 (3.3)	0.968
Hepatomegaly, n (%)	1 (0.4)	1 (0.6)	0	1.000
Splenomegaly, n (%)	7 (2.7)	6 (3.6)	1 (1.1)	0.445
Electrolyte imbalances, n (%)	173 (66.5)	114 (67.5)	59 (64.8)	0.669
Hypoproteinemia, n (%)	95 (36.5)	58 (34.3)	37 (40.7)	0.369
Hepatic dysfunction, n (%)	105 (40.4)	74 (43.8)	31 (34.1)	0.128
Renal dysfunction, n (%)	35 (13.5)	19 (11.2)	16 (17.6)	0.139
Pneumonia, n (%)	33 (12.7)	16 (9.5)	17 (18.7)	0.033
Patients did Weil-Felix test, n (%)	196 (75.4)	105 (62.1)	91 (100)	<0.001
Leucopoenia, n (%)	28 (10.8)	21 (12.4)	7 (7.7))	0.245
Thrombocytopenia, n (%)	65 (25.0)	49 (29.0)	16 (17.6)	0.053
Anemia, n (%)	138 (53.1)	90 (53.3)	48 (52.7)	0.917
Use of macrolides, n (%)	214 (82.3)	139 (82.2)	75 (82.4)	0.973
Use of chloramphenicol, n (%)	30 (11.5)	17 (10.1)	13 (14.3)	0.309
Use of tetracycline antibiotics, n (%)	16 (6.2)	13 (7.7)	3 (3.3)	0.160
Duration of antibiotic, days	5 (5-7)	5 (5-7)	5 (5-7)	0.175
LOS, days	6 (5-8)	6 (5-8)	7 (6-8)	0.007
Cost, CNY, 10^3	2.6 (1.9-3.8)	2.9 (1.7-3.8)	2.6 (1.8-3.7)	0.767
Known fetal outcome, n (%)	238 (91.5)	158 (93.5)	80 (87.9)	0.123
Total fetal loss, n (%)	57 (23.9)	35 (22.2)	22 (27.5)	0.361
Miscarriage, n (%)	36 (15.1)	23 (14.6)	13 (16.3)	0.731
Fetal death/Stillbirth, n (%)	21 (8.8)	12 (7.6)	9 (11.3)	0.348
Preterm birth, n (%)	33 (13.9)	18 (11.4)	15 (18.8)	0.121
Poor fetal outcome, n (%)	90 (37.8)	53 (33.5)	37 (46.3)	0.056

Abbreviations: ST, scrub typhus; T, temperature; LOS, length of hospitalization; CNY, Chinese Yuan (7CNY=1USD). Values are given in n (%), or median and interquartile range (IQR). Chi square or Fisher's exact test were used for categorical variables, and Mann-Whitney U test was applied for comparisons of continuous variables

Conclusions
-Are the conclusions supported by the data presented?
-Are the limitations of analysis clearly described?
-Do the authors discuss how these data can be helpful to advance our understanding of the topic under study?
-Is public health relevance addressed?

Reviewer #1: Please add ICU / hdu admissions also.ST with pneumonia frequently need icu admissions. Add this important finding also could not understand the rationale of using chloramphenicol in pregnancy, please elaborate. Thanks

Reply: Thank you for the suggestion.

We add the information of maternal outcome. There was no maternal death due to the infection, but 3.1% (n=8) admitted to ICU, including 1 complicated with pneumonia and heart failure, 1 with pneumonia and pleural effusion, 1 with pneumonia and septic shock, 1 with septic shock, 1 with pleural effusion, 1 with heart failure and 2 with DIC, in which 2 received chloramphenicol. Please find the revision in lines 337-341 in the file labeled 'Revised Manuscript with Track Changes-PNTD-D-24-01393'.

We agree that the use of chloramphenicol in pregnant women with ST should be avoided as much as possible. Obviously, our results did show the use of chloramphenicol was associated with a poor fetal outcome in univariate logistic-regression analysis with $p<0.05$ and pneumonia was an independent risk factor for a poor fetal outcome on multivariate logistic-regression analysis with an odds ratio of 3.1 (95% CI 1.3-7.6, p=0.011).

In this retrospective review it was not possible to accurately document the indication for the use of chloramphenicol in pregnancy, but we found the use of chloramphenicol decreased significantly, and the proportion of poor fetal outcomes was relatively lower in the latter half of our observation period.

In lines 471-475 in discussion in the file labeled 'Revised Manuscript with Track Changes-PNTD-D-24-01393', we also mentioned: "Unfortunately, the management of ST remains controversial, and no specific treatment regimen has demonstrated a clear advantage or disadvantage in terms of efficacy or safety [29-30], not to mention the recommendation for pregnant women with ST. Further studies are needed to identify the most appropriate antibiotics for this population".

Reviewer #2: The conclusions are difficult to assess owing to the inadequate confirmation of the diagnosis of scrub typhus.

Reply: Thank you for the suggestion.

We agree that a fourfold rise or greater in titer would be more supportive of the diagnosis than a titer of 1:80. Based on your comments, we revised the laboratory diagnostic criteria: A minimum titer of 1:160 or a fourfold increase over previous levels for OXK is considered diagnostically significant. Please find the revision in line 155 in the file labeled 'Revised Manuscript with Track Changes-PNTD-D-24-01393'.

Based on the revised laboratory diagnostic criteria, we reanalyzed the Weil-Felix test results of all 260 patients. In total, 75.4% (n=196) had Weil-Felix test, in which 52.5%

(n=103), 1.0% (n=2) and 46.4% (n=91) had a titer of 1:40, 1:80, and ≥1:160 for OXK, respectively. Please find the revision in lines 185-187 in the file labeled 'Revised Manuscript with Track Changes-PNTD-D-24-01393'.

In addition, patients were separated into clinically diagnosed cases and laboratory confirmed cases. The clinical presentation, treatment, and outcome were compared for patients between clinically diagnosed cases and laboratory confirmed cases. Please find the revision in lines 184-194 and table 1 in the file labeled 'Revised Manuscript with Track Changes-PNTD-D-24-01393'.

Editorial and Data Presentation Modifications?

Use this section for editorial suggestions as well as relatively minor modifications of existing data that would enhance clarity. If the only modifications needed are minor and/or editorial, you may wish to recommend "Minor Revision" or "Accept".

Reviewer #1: Minor revision.
Reply: Thank you for the positive comments.

Reviewer #2: See below
Reply: See below in details.

Summary and General Comments

Use this section to provide overall comments, discuss strengths/weaknesses of the study, novelty, significance, general execution and scholarship. You may also include additional comments for the author, including concerns about dual publication, research ethics, or publication ethics. If requesting major revision, please articulate the new experiments that are needed.

Reviewer #1: Please add ICU / hdu admissions also.ST with pneumonia frequently need icu admissions. Add this important finding also .could not understand the rationale of using chloramphenicol in pregnancy, please elaborate . Thanks
Reply: Thank you for the suggestion.
We add the information of maternal outcome. There was no maternal death due to the infection, but 3.1% (n=8) admitted to ICU, including 1 complicated with pneumonia and heart failure, 1 with pneumonia and pleural effusion, 1 with pneumonia and septic shock, 1 with septic shock, 1 with pleural effusion, 1 with heart failure and 2 with DIC, in which 2 received chloramphenicol. Please find the revision in lines 337-341 in the file labeled 'Revised Manuscript with Track Changes-PNTD-D-24-01393'.
We agree that the use of chloramphenicol in pregnant women with ST should be avoided as much as possible. Obviously, our results did show the use of chloramphenicol was associated with a poor fetal outcome in univariate logistic-regression analysis with $p<0.05$ and pneumonia was an independent risk factor for a poor fetal outcome on multivariate logistic-regression analysis with an odds ratio of 3.1 (95% CI 1.3-7.6, p=0.011).

In this retrospective review it was not possible to accurately document the indication for the use of chloramphenicol in pregnancy, but we found the use of chloramphenicol decreased significantly, and the proportion of poor fetal outcomes was relatively lower in the latter half of our observation period.

In lines 471-475 in discussion in the file labeled 'Revised Manuscript with Track Changes-PNTD-D-24-01393', we also mentioned: “Unfortunately, the management of ST remains controversial, and no specific treatment regimen has demonstrated a clear advantage or disadvantage in terms of efficacy or safety [29-30], not to mention the recommendation for pregnant women with ST. Further studies are needed to identify the most appropriate antibiotics for this population”.

Reviewer #2: If the authors stratified their results according to confirmed cases vs cases based upon the clinical diagnosis only, this might be a useful contribution of new knowledge.

Reply: Thank you for the suggestion.

Based on your comments, patients were separated into clinically diagnosed cases and laboratory confirmed cases. The clinical presentation, treatment, and outcome were compared for patients between clinically diagnosed cases and laboratory confirmed cases. Please find the revision in lines 184-194 and table 1 in the file labeled 'Revised Manuscript with Track Changes-PNTD-D-24-01393'.

A total of 260 pregnant women were diagnosed with ST, including clinically diagnosed cases 65.0% (n=169) and laboratory confirmed cases 35.0% (n=91) (Table 1). There were no statistically significant differences in antibiotic treatment and fetal outcome, but a higher proportion of patients with pneumonia (18.7% vs. 9.5%, p=0.033) and a longer length of hospitalization (7 vs. 6 days, p=0.007) were seen in laboratory confirmed cases than clinically diagnosed cases.

Table 1 Clinically diagnosed cases Versus laboratory confirmed cases: clinical profile, management and outcome of 260 pregnant women with ST

Diagnosis	Total	clinically diagnosed	laboratory confirmed	P value
N of patients, n (%)	260	169 (65.0)	91 (35.0)	-
Pregnancy in weeks	24 (18-30)	24 (18-32)	24 (19-28)	0.471
First trimester, n (%)	39 (15.0)	24 (14.2)	15 (16.5)	0.623
Second trimester, n (%)	121 (46.5)	76 (45.0)	45 (49.5)	0.490
Third trimester, n (%)	100 (38.5)	69 (40.8)	31 (34.1)	0.285
T $\geq$ 39°C, n (%)	165 (63.5)	113 (66.9)	52 (57.1)	0.235
Duration of fever, days	6 (4-8)	6 (5-8)	6 (4-8)	0.453
Eschar, n (%)	204 (78.5)	169 (100)	35 (38.5)	<0.001
Lymphadenopathy, n (%)	10 (3.8)	5 (3.0)	5 (5.5)	0.499
Skin rash, n (%)	7 (2.7)	4 (2.4)	3 (3.3)	0.968
Hepatomegaly, n (%)	1 (0.4)	1 (0.6)	0	1.000
Splenomegaly, n (%)	7 (2.7)	6 (3.6)	1 (1.1)	0.445
Electrolyte imbalances, n (%)	173 (66.5)	114 (67.5)	59 (64.8)	0.669
Hypoproteinemia, n (%)	95 (36.5)	58 (34.3)	37 (40.7)	0.369
Hepatic dysfunction, n (%)	105 (40.4)	74 (43.8)	31 (34.1)	0.128
Renal dysfunction, n (%)	35 (13.5)	19 (11.2)	16 (17.6)	0.139
Pneumonia, n (%)	33 (12.7)	16 (9.5)	17 (18.7)	0.033
Patients did Weil-Felix test, n (%)	196 (75.4)	105 (62.1)	91 (100)	<0.001
Leucopoenia, n (%)	28 (10.8)	21 (12.4)	7 (7.7))	0.245
Thrombocytopenia, n (%)	65 (25.0)	49 (29.0)	16 (17.6)	0.053
Anemia, n (%)	138 (53.1)	90 (53.3)	48 (52.7)	0.917
Use of macrolides, n (%)	214 (82.3)	139 (82.2)	75 (82.4)	0.973
Use of chloramphenicol, n (%)	30 (11.5)	17 (10.1)	13 (14.3)	0.309
Use of tetracycline antibiotics, n (%)	16 (6.2)	13 (7.7)	3 (3.3)	0.160
Duration of antibiotic, days	5 (5-7)	5 (5-7)	5 (5-7)	0.175
LOS, days	6 (5-8)	6 (5-8)	7 (6-8)	0.007
Cost, CNY, 10^3	2.6 (1.9-3.8)	2.9 (1.7-3.8)	2.6 (1.8-3.7)	0.767
Known fetal outcome, n (%)	238 (91.5)	158 (93.5)	80 (87.9)	0.123
Total fetal loss, n (%)	57 (23.9)	35 (22.2)	22 (27.5)	0.361
Miscarriage, n (%)	36 (15.1)	23 (14.6)	13 (16.3)	0.731
Fetal death/Stillbirth, n (%)	21 (8.8)	12 (7.6)	9 (11.3)	0.348
Preterm birth, n (%)	33 (13.9)	18 (11.4)	15 (18.8)	0.121
Poor fetal outcome, n (%)	90 (37.8)	53 (33.5)	37 (46.3)	0.056

Abbreviations: ST, scrub typhus; T, temperature; LOS, length of hospitalization; CNY, Chinese Yuan (7CNY=1USD). Values are given in n (%), or median and interquartile range (IQR). Chi square or Fisher's exact test were used for categorical variables, and Mann-Whitney U test was applied for comparisons of continuous variables
